# Multivalent cations stabilize DNA duplexes beyond charge neutralization

**DOI:** 10.1039/d6sc03808e

**Published:** 2026-09-01

**Authors:** Yun-Long Chen, Chen-Chen Zheng, Yan Zhang, Zhi-Jie Tan, Xing-Hua Zhang

**Affiliations:** a Hubei Key Laboratory of Cell Homeostasis, College of Life Sciences, School of Physics and Technology, Women and Children's Hospital, Renmin Hospital of Wuhan University, Wuhan University Wuhan 430072 China zjtan@whu.edu.cn zhxh@whu.edu.cn; b School of Physics and Optical Engineering, Zhejiang University of Technology Hangzhou 310023 China; c Department of Clinical Laboratory, Renmin Hospital of Wuhan University Wuhan China peneyyan@whu.edu.cn

## Abstract

Multivalent cations are abundant in cells and play essential roles in DNA duplex stability, genome packaging, and DNA–protein interactions. They can also condense DNA, making it challenging to determine their influence on DNA duplex stability. To overcome this challenge, we studied DNA unpeeling at equilibrium under high tension using magnetic tweezers, thereby preventing condensation. Experiments show that DNA duplex stability first increases and then decreases as cation concentration increases and the maximum DNA duplex stability increases with cation valence. The maximum free energy change of DNA was 3.33 *k*_B_*T*/bp for Na^+^ and increased to 3.98 *k*_B_*T*/bp for protamine, which is a small arginine-rich protein with a highly positive charge (≈21 for salmon sperm), corresponding to a relative increase of 19.5%. Consistently, all-atom molecular dynamics simulations show that higher-valent cations preferentially embed in the minor groove of DNA and clamp the minor groove, in contrast to the major-groove clamping reported for RNA, thereby stabilizing the helix more efficiently. These findings establish a single-molecule framework for quantifying DNA thermodynamics in complex ionic environments, which contributes to understanding ionic control of genome stability and to designing ion-tunable DNA-based nanostructures and delivery systems.

## Abstract 2

High-tension single-molecule melting suppresses DNA condensation and reveals a valence-dependent increase in DNA stability, explained by multivalent-cation clamping of the DNA minor groove.

## Graphical abstract

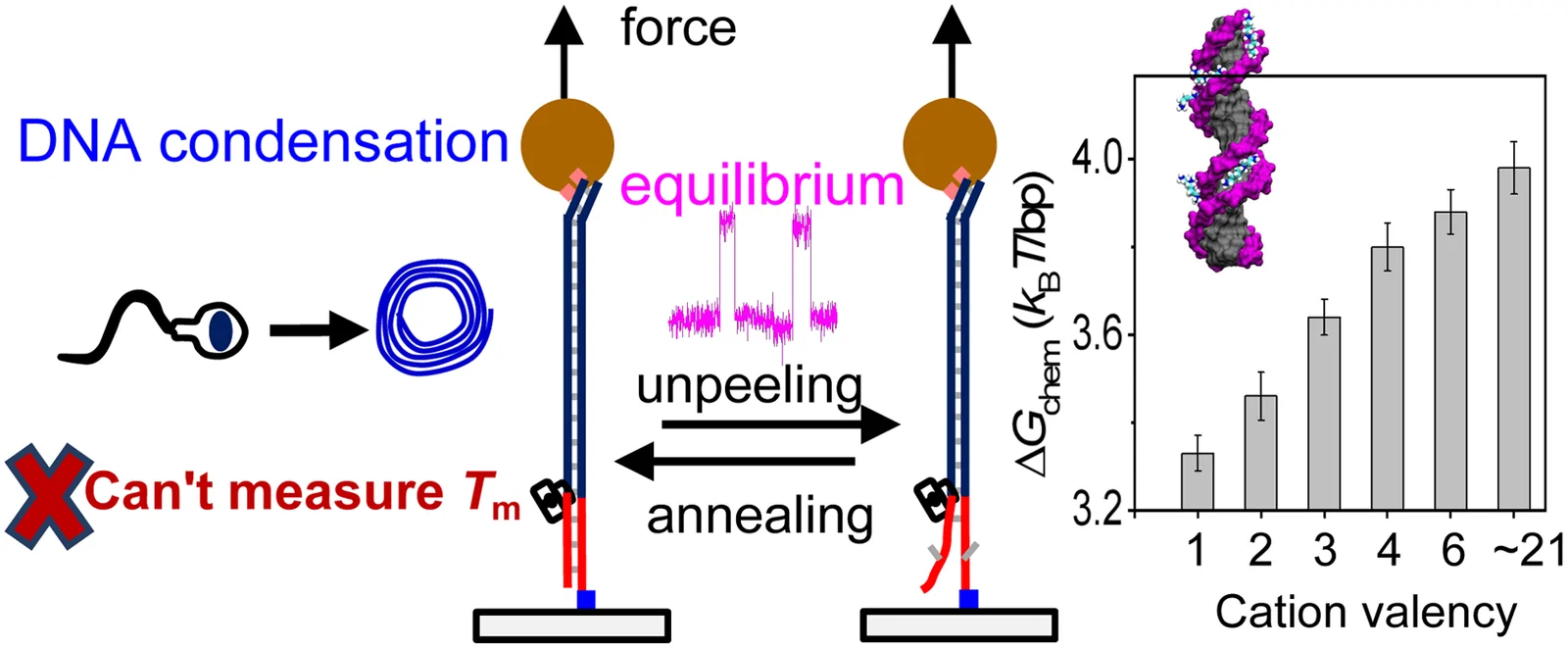

## Introduction

Double-stranded DNA (dsDNA) consists of two complementary strands, which melt into two single-stranded DNA (ssDNA) during fundamental biological processes such as replication, transcription, and repair by enzymes such as polymerases and helicases.^1–4^ DNA duplex stability governs these biological functions and is also important in the rational engineering of DNA nanostructures.^5–7^ DNA duplex stability depends on factors such as salt, temperature, and sequence.^4,8–10^ Ions mitigate the strong electrostatic repulsion of the phosphodiester backbone by screening negative charges, so that increasing ionic strength raises duplex stability.

Multivalent cations are abundant in cells and modulate the DNA structure and stability, and they also promote genome packaging through DNA condensation. DNA condensation refers to the process in which negatively charged DNA molecules are compacted into highly ordered and dense structures through charge neutralization.^11,12^ Representative cellular or biomimetic high-valent cations include spermidine (SPD^3+^) and spermine (SM^4+^), which function as protective agents and modulators of the DNA secondary structure.^13^ Polyamine–DNA interactions have long been studied. Early studies showed that spermine and other polyamines protect DNA against heat denaturation,^13^ and molecular models proposed that polyamines bind along DNA grooves and phosphate groups to stabilize the duplex.^14–16^ We also studied the polylysine peptide hexa-lysine (PLL^6+^), commonly employed as a reduced model of the highly charged, intrinsically disordered, lysine-rich histone tails,^17,18^ and protamine,^19,20^ a small arginine-rich protein with a highly positive charge (average net charge ≈ 21+ for salmon sperm protamine).

Understanding these effects is important for elucidating the physical basis of DNA condensation and duplex denaturation.^11,12^*In vitro*, high-resolution melting (HRM) is a commonly used tool for analyzing DNA duplex stability by measuring the melting temperature (*T*_m_).^21–25^ Previous studies using the Peyrard–Bishop–Dauxois model also showed that salt can increase both *T*_m_ and unzipping force by screening phosphate–phosphate repulsion,^10,26^ but intrinsic duplex stability under strongly condensing multivalent cation conditions remains difficult to quantify experimentally.

Recent rapid development of single-molecule techniques has made it possible to study DNA duplex stability with high precision.^27–32^ In single-DNA manipulation experiments, a DNA molecule is tethered between a surface (*e.g.*, a coverslip) at one end and a probe (*e.g.*, a bead) at the other end, and a mechanical constraint such as tensile force is applied through the probe. The mechanical overstretching of DNA occurs at ∼65 pN and increases the contour length by ∼70% compared with B-DNA.^29^ Besides the B-to-S transition, unpeeling is another DNA overstretching pathway that initiates at nicks or free ends and produces ssDNA under tension when base pairing is weak due to high temperature, low ionic strength, or low GC content.^29–32^ During unpeeling, the applied force can prevent DNA condensation in the presence of multivalent cations, enabling DNA melting to be detected under such conditions.

In this work, we explored the effects of cations (Na^+^, Ca^2+^, SPD^3+^, SM^4+^, PLL^6+^, and protamine as examples) on DNA duplex stability by unpeeling at equilibrium using single-molecule magnetic tweezers (MT) and performed complementary all-atom molecular dynamics (MD) simulations to examine cation binding and the structural response of DNA, providing mechanistic insight. These results help us to evaluate how these cations affect the stability and functions of DNA.

## Experimental

### HRM experiments

**Fig. 1:**
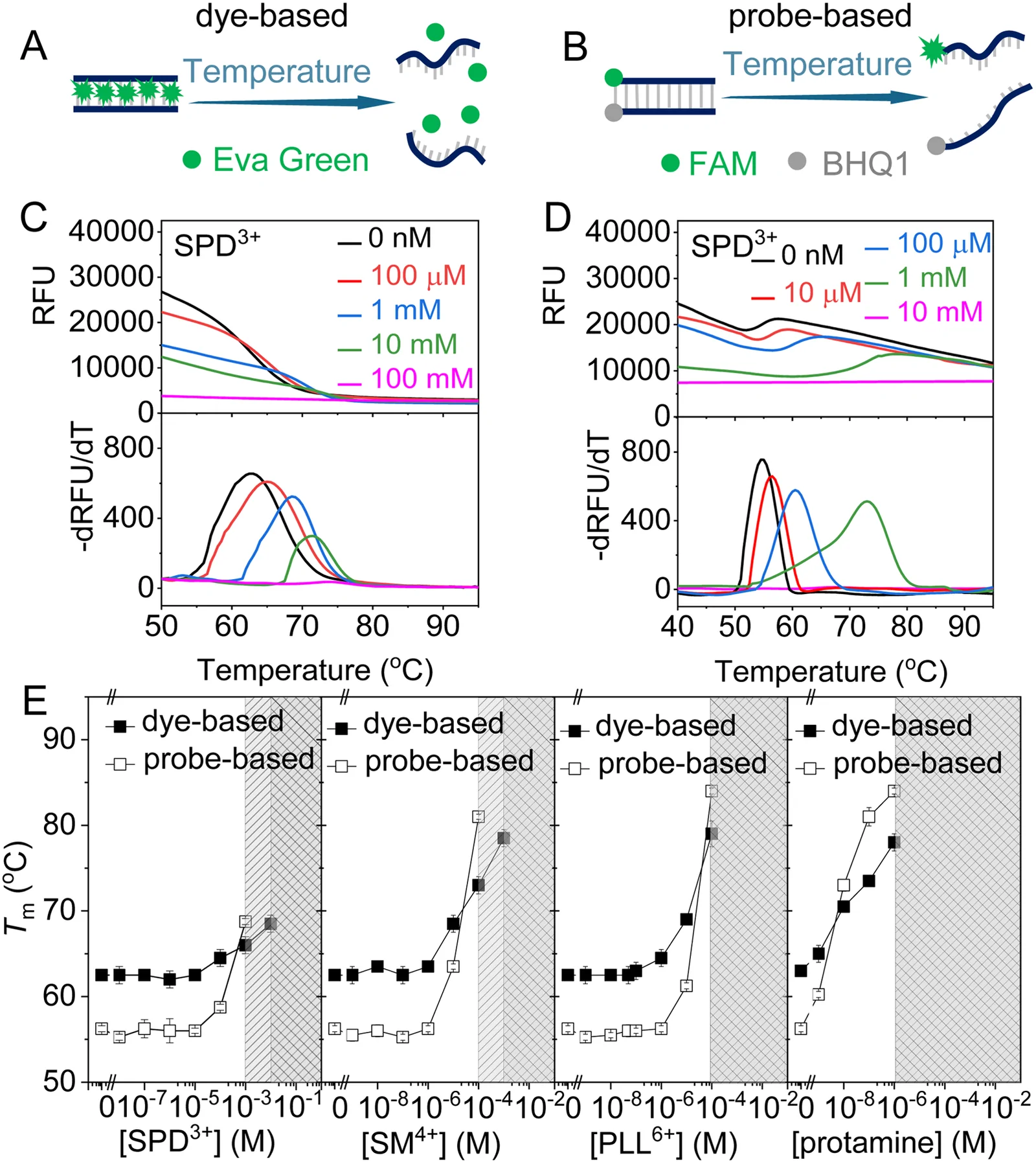
*T*_m_ *versus* multivalent cation concentration measured by HRM assays. (A and B) Schematic diagram of the dye-based and probe-based HRM assays. (C and D) Representative fluorescence response curves and their negative first derivatives (−dRFU/d*T*) for dsDNA at different SPD^3+^ concentrations, measured with the dye-based and probe-based assays, respectively. RFU represents the relative fluorescence unit. (E) *T*_m_ of DNA as a function of multivalent cation concentrations. Shaded areas denote cation concentrations at which *T*_m_ could not be determined.

We purchased spermidine·3HCl, spermine·4HCl, and protamine sulfate from Sigma-Aldrich. Hexa-lysine·6HCl was synthesized by Sangon Biotech.^17^ For HRM analysis, two complementary 22-nt ssDNA strands (5′-GACGATCGTTCGTGAAGTCAAC-3′, ssDNA1 and 5′-GTTGACTTCACGAACGATCGTC-3′, ssDNA2) were annealed from 70 °C to 10 °C at a rate of 1 °C min to form a 22-bp dsDNA. Each 20 µL reaction mixture contained 5 µM DNA and a certain concentration of multivalent cation in 10 mM Tris–HCl (pH 7.5). For dye-based HRM, EvaGreen (100 µM), a third-generation intercalating dye, was used (Fig. 1A and S1). For probe-based HRM without DNA dye, one strand was labeled at the 5′ end with FAM and the complementary strand was labeled with BHQ1 at the 3′ end (Fig. 1B and S2). In addition, we measured the fluorescence response of two 22-nt ssDNA strands inside the 22-bp dsDNA separately as controls under the same dye-based HRM conditions. We also performed dye-based HRM experiments at different dsDNA concentrations (0.5, 5, 20, and 50 µM dsDNA) using SM^4+^ as a representative strongly condensing multivalent cation. All reactions were performed using a real-time PCR system in 96-well plates. The melting protocol consisted of a 60 s hold at 40 °C, followed by melting from 45 °C to 95 °C in 0.5 °C increments every 5 s with real-time fluorescence acquisition. Data were analyzed using Bio-Rad Precision Melt Analysis Software v1.2.^22^

### MT experiments

**Fig. 2:**
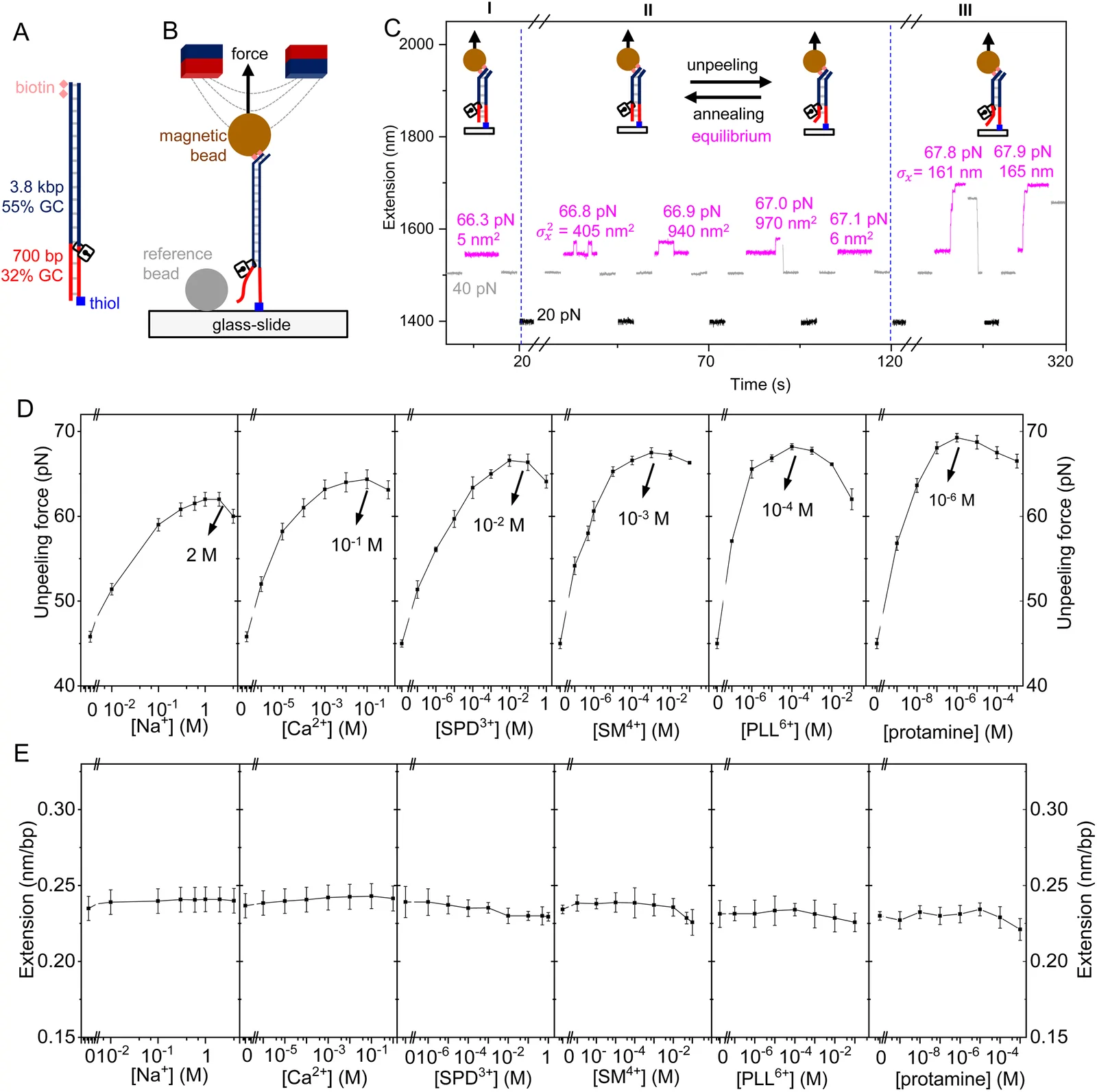
Determination of the forces and extension change during unpeeling by repeating force-cycles. (A) The DNA construct. The DNA comprises two segments with different GC contents (a 4.3-kbp region with 55% GC and a 0.7-kbp region with 32% GC), joined by a 21-bp GC-rich core that blocks unpeeling. (B) MT setup. The DNA was anchored between a superparamagnetic microbead and a coverslip. (C) A typical time-extension course recorded during repeating force-cycles at 0.1 mM PLL^6+^. In region I, the DNA was not unpeeled. In region II, DNA began to unpeel, and we identified the unpeeling force at the onset at which the extension variance (*σ*_*x*_^2^) exceeded 100 nm^2^. In region III, the AT-rich segment of the DNA duplex was completely unpeeled, allowing the calculation of the extension increase during unpeeling (*σ*_*x*_). (D and E) Force and extension change during DNA unpeeling at different concentrations of multivalent cations.

In MT experiments, a 5-kbp dsDNA construct was prepared by PCR using a multi-biotin-labeled primer and a single-thiol-labeled primer.^33^ The DNA construct comprised two segments with different GC contents (a 4.3-kbp region with 55% GC and a 0.7-kbp region with 32% GC, Fig. 2A and B), joined by a 21-bp GC core (CGGCCCGGGGGCCCCGGCGG). Detailed construct preparation is provided in the SI (Fig. S3 and Method S1). The thiol-labeled end of the DNA molecule was anchored to a sulfo-SMCC-coated (Sigma) coverslip and then the multiply biotin-labeled end of the DNA molecule was anchored to a streptavidin-coated superparamagnetic microbead (M-270, Dynabeads). A pair of NdFeB magnets was used to attract the bead and exert constant forces on the anchored DNA molecule. The applied force was controlled by the position of the magnets, and the value of the applied force was calculated based on the Brownian motion of the bead.^32^ Please see more details about the DNA unpeeling measurements in Fig. 2C.

### MD experiments

In all-atom MD simulations, a 20-bp dsDNA (CGACTCTACGGCATCTGCGC) was constructed with the Nucleic Acid Builder package^34^ and was solvated in explicit water with added ions to neutralize the negative charge of the dsDNA.^35–38^ The 20-bp DNA used in the simulations was chosen as a generic, non-designed B-form duplex rather than a sequence with known unusual groove geometry; nevertheless, special sequences that generate distinct groove widths or local electrostatic potentials may quantitatively affect cation binding, groove narrowing, and clamping.^15,39^ We performed a series of all-atom MD simulations under salt conditions including 1000 mM Na^+^, 100 mM Ca^2+^, 2 mM SPD^3+^, 0.1 mM PLL^6+^ and 1 µM salmon protamine.^40^ The initial protamine structure was generated with tleap in AmberTools^41^ and parameterized using ff14SB.^42^ Due to the large size of protamine, the chain was divided into three segments to localize its binding positions on DNA, with each segment containing 11 residues. The interactions of each segment with DNA were then analyzed separately using the same distance criterion. All-atom MD simulations were performed in GROMACS 2021.5 (ref. 43) using AMBER ff99bsc1 for dsDNA^44^ and TIP3P water,^45,46^ and long-range electrostatic interactions were computed using the particle mesh Ewald method.^47^ After standard energy minimization and equilibration, production runs (600 ns per condition) were carried out, and equilibrated trajectories were used for analysis (Fig. S4). Further details are available in our previous studies^48–51^ and the SI. Helical parameters (including helical rise and major/minor groove widths) were analyzed with Curves+ after three base pairs at each end were removed to avoid end effects.^52^ Ion binding was quantified by defining binding ions as those within 6 Å of phosphate groups and clamping ions as those that simultaneously lie within 6 Å of both DNA strands, and clamping ions were further classified as major- or minor-groove clamping ions based on their locations.^49,50,53–56^ Full details of system setup, ion models, parameterization, simulation protocols, and analysis are provided in the SI (Method S2).

## Results and discussion

### HRM cannot determine multivalent cation effects on *T*_m_

We first employed the dye-based HRM assay to investigate the effects of multivalent cations (including SPD^3+^, SM^4+^, PLL^6+^, and protamine) on DNA duplex stability. As shown in Fig. 1C and E, *T*_m_ increased with increasing multivalent-cation concentrations. However, the melting curves flattened (Fig. 1C and S1) and *T*_m_ could not be obtained at higher cation concentrations (shaded area in Fig. 1E) due to DNA condensation.

To avoid potential dye-induced bias, we used a probe-based HRM assay in which a fluorophore-quencher-labeled duplex shows an increase in fluorescence upon melting (Fig. 1B). However, this assay also failed to yield *T*_m_ at high concentrations of multivalent cations (shaded region in Fig. 1E) as the fluorescence signal remained low and changed little during heating (Fig. 1D and S2). Therefore, neither dye-based nor probe-based HRM can reliably assess the effects of multivalent cations on DNA duplex stability.

Interestingly, *T*_m_ obtained from dye-based and probe-based HRM assays exhibited distinct dependencies on multivalent-cation concentration. In the absence of cations, the dye-based HRM yielded a higher *T*_m_, which may be due to dye binding stabilizing DNA. However, as the concentration of multivalent cations increased, *T*_m_ yielded from the dye-based HRM was lower than that from the probe-based HRM, which may be because the cations altered the binding of the dye to DNA. Such an opposite trend of measured *T*_m_ was also observed for monovalent salts such as NaCl and LiCl in our previous work.^57^

To further study whether cation-mediated intermolecular interactions between dsDNA can have an influence on the measured *T*_m_ of dsDNA, we separately measured the fluorescence responses of the two 22-nt ssDNA strands corresponding to the 22-bp dsDNA under the same HRM conditions as controls. As shown in Fig. S5–S8, the fluorescence signals from the individual ssDNA strands had only a minor effect on the measured *T*_m_ of the dsDNA. In addition, the cation-mediated intermolecular interactions between dsDNA molecules may influence the *T*_m_ by stabilizing the duplexes through the formation of higher-order structures, and their contribution would therefore be expected to depend on the concentration of dsDNA. Thus, we performed HRM experiments at different dsDNA concentrations (0.5, 5, 20 and 50 µM dsDNA) (Fig. S9). We found that *T*_m_ increased with dsDNA concentration under all conditions, as expected for bimolecular duplex melting, but the concentration dependence became more pronounced in the presence of SM^4+^. This additional dependence is consistent with a contribution from SM^4+^-mediated intermolecular association or higher-order structures. Therefore, we designed force-induced dsDNA unpeeling experiments, which can measure the duplex stability of single dsDNA molecules and effectively avoid interference from multivalent-cation-induced higher-order DNA structures.

### Force-induced unpeeling determines multivalent cation effects on DNA duplex stability

To overcome the challenge of determining multivalent cation effects on DNA duplex stability, we studied force-induced DNA unpeeling at equilibrium under high tension using MT experiments. We used a 5-kbp dsDNA construct containing a low-GC segment and a high-GC segment separated by a highly stable 21-bp GC island, which blocks unpeeling (Fig. 2A and B). Because multiple biotin–streptavidin attachments at the bead-proximal end are located within the biotin-labeled DNA segment, its terminal duplex lies outside the force-bearing path, whereas the coverslip-proximal AT-rich end is tethered through a single terminal thiol, allowing the applied force to reach the duplex end and initiate unpeeling there. We performed the MT measurements under very low DNA-tethering-density conditions, typically with only one DNA-tethered bead in each field of view. We removed free DNA by buffer exchange before the unpeeling measurements. We thereby avoided possible interference from one-end-tethered DNA molecules^58^ and free neighboring dsDNA duplexes in solution. More details about the preparation of DNA can be found in the SI (Fig. S3 and Method S1).

We performed repeated force-cycles (Fig. 2C). Each cycle included three force levels: low (∼20 pN), intermediate (∼45 pN), and high, at which the force was increased incrementally by ∼0.1 pN in each cycle. A representative time-extension trace is shown in Fig. 2C, which can be divided into three time-ordered regions: In region I, DNA duplex remained fully hybridized even at high forces. In region II, DNA began to unpeel, and we identified the unpeeling force at the onset where the extension variance exceeded 100 nm^2^. In this region, we could determine the equilibrium unpeeling force indicated by the hopping kinetics of DNA extension. Note that, after unpeeling, one ssDNA strand remains under tension, whereas in thermal melting both separated strands are force-free. In region III, the AT-rich segment of the DNA duplex was completely unpeeled, allowing calculation of the extension increase per base pair during unpeeling. Notably, the force at which DNA was completely unpeeled was only slightly higher than that of the unpeeling onset (67.8 pN compared to 66.8 pN in this example).

As shown in Fig. 2D, the unpeeling force exhibited a non-monotonic dependence on cation concentration: it increased to a maximum and then decreased. The concentrations at which the reversal occurred were 2 M for Na^+^, 0.1 M for Ca^2+^, 10 mM for SPD^3+^, and 1 mM for SM^4+^, which are consistent with our previously reported DNA charge inversion thresholds.^18,57^ Thus, we attribute the reversal in unpeeling force to DNA charge inversion caused by the excessive binding of multivalent cations.^12^ Based on the measured unpeeling force and the corresponding DNA extension increase during unpeeling (Fig. 2D and E), we calculated the equilibrium chemical free energy of DNA duplex dissociation using the theoretical framework of Rouzina and Bloomfield:^53^

1 Δ*G* = Δ*G*_chem_ − *F*_e_·Δ*l* = 0

Here, Δ*G* is the total free energy change per base pair during unpeeling; Δ*G*_chem_ is the ionic-strength-dependent free energy change of DNA; *F* is the equilibrium unpeeling force (Fig. 2D); Δ*l* is the change of DNA extension per base pair at *F*_e_ (Δ*l* = *σ*_*x*_/700, Fig. 2E).

**Fig. 3:**
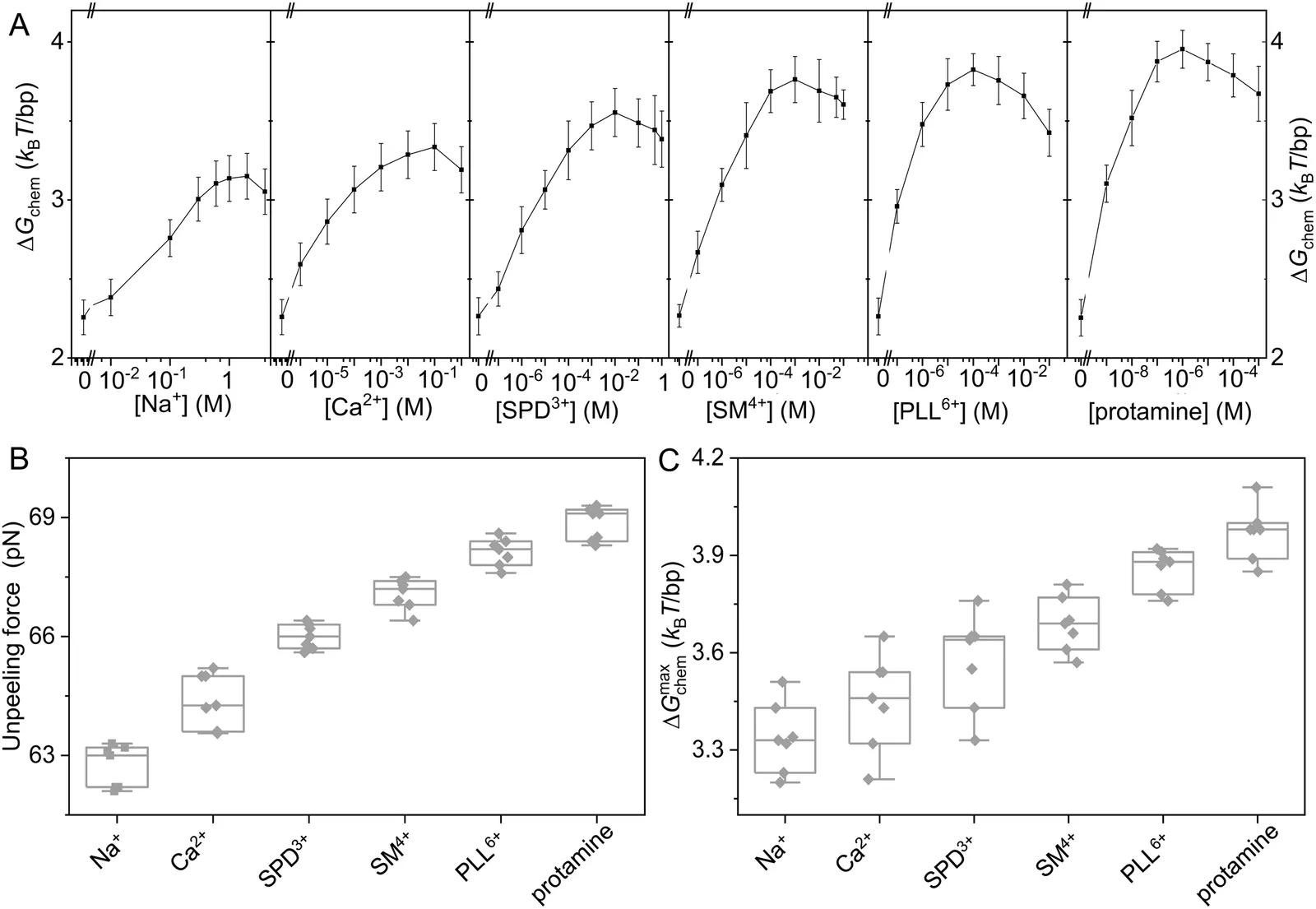
Unpeeling force and free energy change during DNA unpeeling at different concentrations of multivalent cations. (A) Free energy change during DNA unpeeling at different concentrations of multivalent cations. (B) Unpeeling force during DNA unpeeling at the highest DNA duplex stability achieved. (C) Free energy change during DNA unpeeling at the highest DNA duplex stability achieved.

The values of Δ*G* showed a clear non-monotonic dependence of DNA duplex stability on multivalent cation concentration, which may arise from charge inversion indicated by the concentration for each type of salt (Fig. 3A).^12,57,59^ Notably, higher-valent cations stabilize DNA more efficiently, as reflected by the monotonic increase in both *F*^max^_e_ and Δ*G*^max^_chem_ with cation valency (Fig. 3B and C). As shown in Fig. 3B, the *F*^max^_e_ was ∼64.52 pN for Na^+^, ∼65.21 pN for Ca^2+^, ∼66.7 pN for SPD^3+^, ∼67.5 pN for SM^4+^, ∼68.2 pN for PLL^6+^, and ∼69.2 pN for protamine. As shown in Fig. 3C, the Δ*G*^max^_chem _was 3.33 *k*_B_*T*/bp for Na^+^, ∼3.46 *k*_B_*T*/bp for Ca^2+^, ∼3.64 *k*_B_*T*/bp for SPD^3+^, ∼3.80 *k*_B_*T*/bp for SM^4+^, ∼3.88 *k*_B_*T*/bp for PLL^6+^, and ∼3.98 *k*_B_*T*/bp for protamine. The DNA duplex stability, indicated by *F*^max^_e _and Δ*G*^max^_chem_, showed a clear monotonic increase with cation valency: Na^+^ < Ca^2+^ < SPD^3+^ < SM^4+^ < PLL^6+^ < protamine.

### Effects of multivalent cations on the structure and ion binding for DNA from MD simulations

**Fig. 4:**
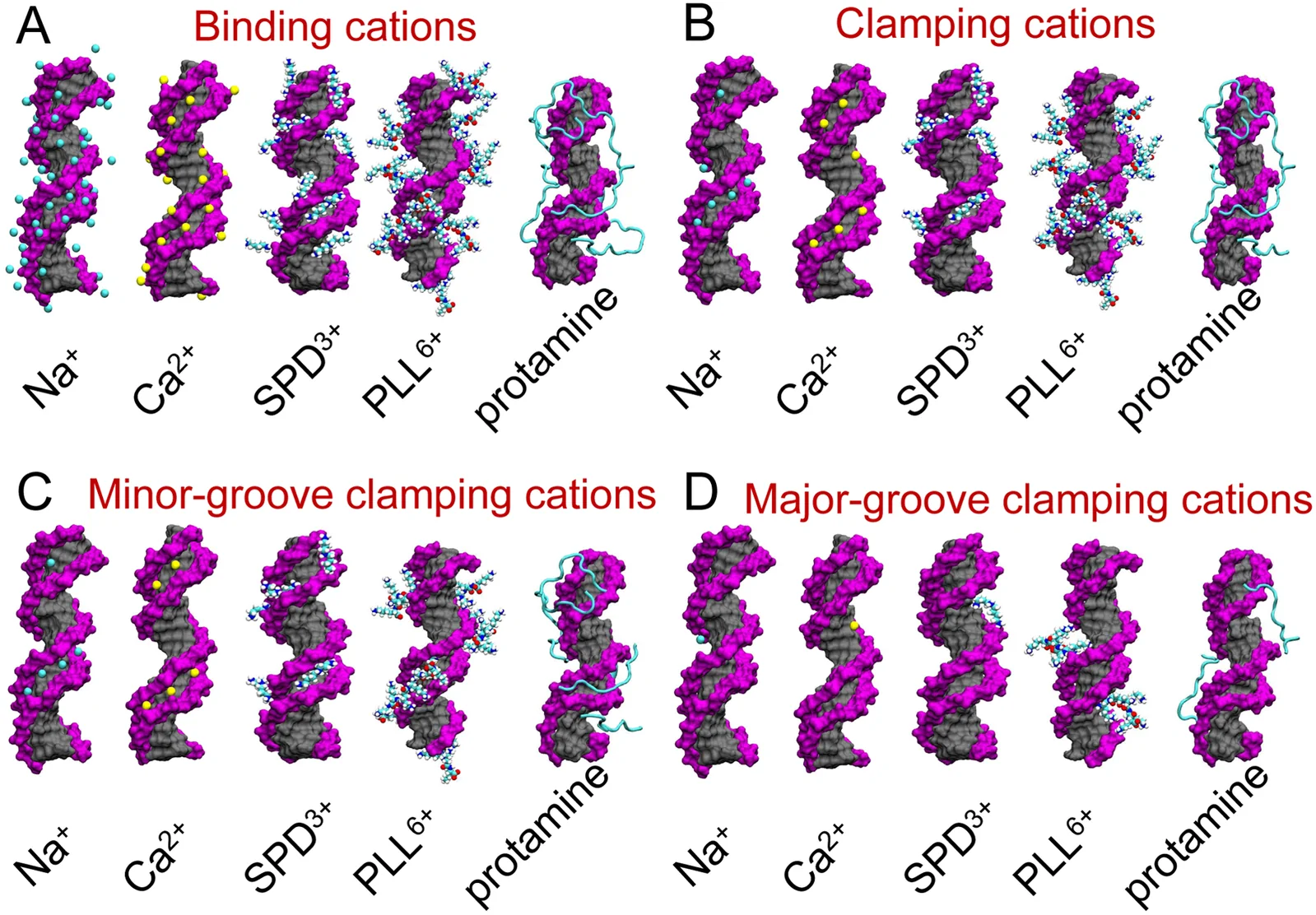
Cations binding to dsDNA obtained from MD simulations. The near-neutralization concentration was used for each type of cation, Na^+^ (1 M), Ca^2+^ (100 mM), SPD^3+^ (2 mM), PLL^6+^ (0.1 mM) and protamine (1 µM). (A) Representative distribution of cations bound around the dsDNA duplex. (B) Only clamping cations are shown for clarity. (C) Cations clamping within the minor groove. (D) Cations clamping within the major groove.

**Fig. 5:**
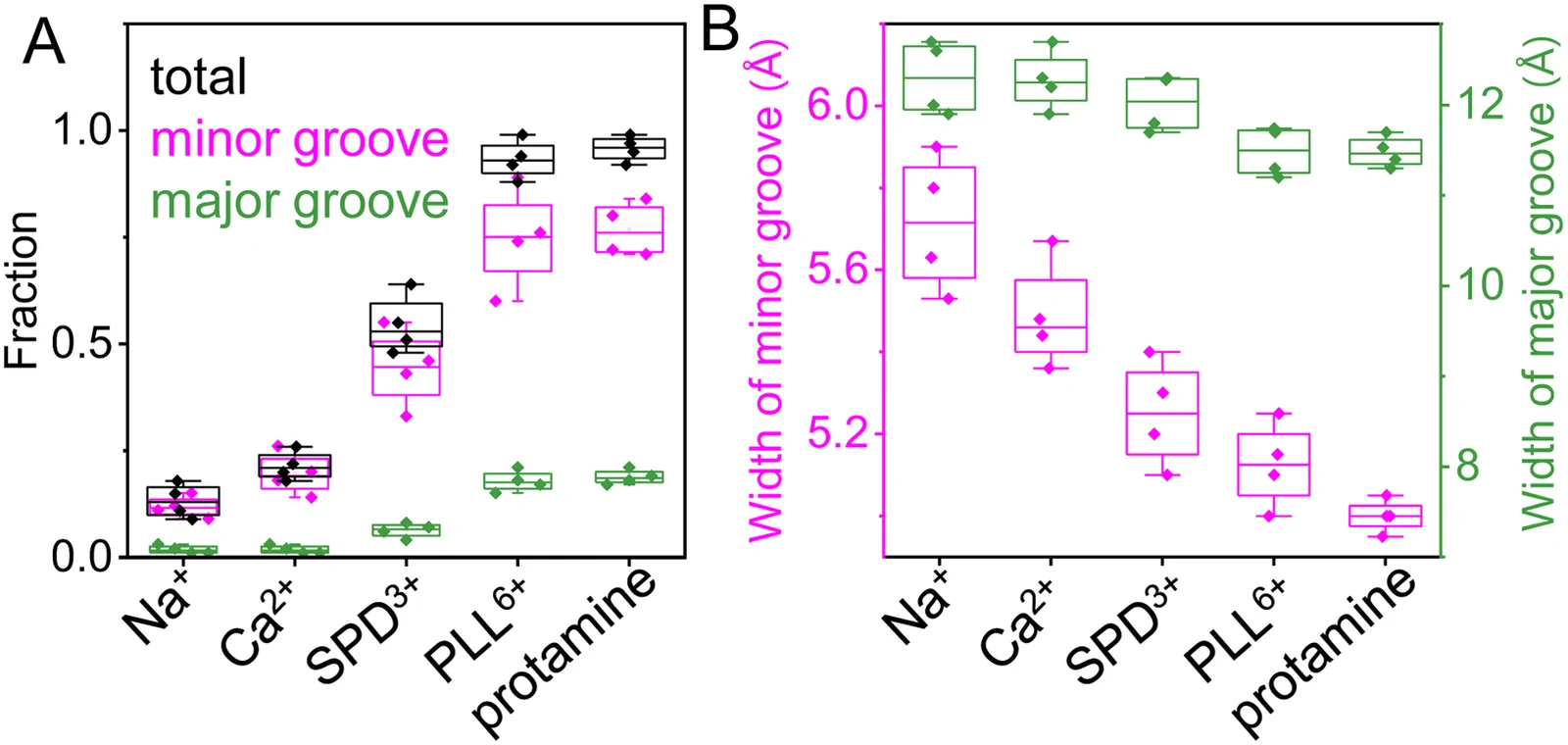
Fraction of clamping cations among all bound cations and effects of cations on the groove width of DNA. (A) Fraction of total (black), minor-groove (magenta) and major-groove (green) clamping cations relative to all bound cations. (B) Cation-driven width changes of the DNA minor (magenta) and major (green) grooves.

To reveal why higher-valent cations stabilize DNA more efficiently, we performed all-atom MD simulations at near-neutralization concentration for each cation (Fig. 4A–D), which is 1 M for Na^+^, 100 mM for Ca^2+^, 2 mM for SPD^3+^, 0.1 mM for PLL^6+^, and 1 µM protamine. The neutralization concentrations were selected to compare different cations at a similar overall DNA charge-compensation state, where cation binding is sufficient for structural analysis but strong overcharging effects are minimized. Each concentration condition was simulated for 600 ns, and the equilibrated trajectories were used for analysis (Fig. S4). We observed that cations embed within DNA grooves and clamp the duplex by attracting both strands (Fig. 5). We define clamping cations in the minor grooves as those that simultaneously approach the two DNA strands and form bridging contacts between them and thus the minor groove is narrowed and its fluctuation is suppressed. As shown in Fig. 5A, the fraction of total clamping cations relative to all bound cations is 0.15 ± 0.06 for Na^+^, 0.20 ± 0.06 for Ca^2+^, 0.51 ± 0.13 for SPD^3+^, 0.94 ± 0.04 for PLL^6+^, and 0.97 ± 0.02 for protamine which increases monotonically with cation valency, consistent with our experimental trend that DNA duplex stability increases with cation valency.

For dsRNA, previous studies have reported that multivalent cations preferentially embed within the major groove and narrowed it through the clamping effect.^60–62^ For dsDNA, here we instead report that cations preferentially embed in the minor groove (Fig. 5A). As shown in Fig. 5A (magenta), the fraction of minor-groove clamping cations relative to all bound cations is 0.15 ± 0.06 for Na^+^, 0.20 ± 0.06 for Ca^2+^, 0.43 ± 0.13 for SPD^3+^, 0.74 ± 0.12 for PLL^6+^, and 0.75 ± 0.15 for protamine. Correspondingly, the minor groove width was 5.63 ± 0.59 Å for Na^+^ and shrank to 5.48 ± 0.57 Å for Ca^2+^, 5.30 ± 0.54 Å for SPD^3+^, 5.15 ± 0.49 Å for PLL^6+^, and 5.03 ± 0.30 Å for protamine (Fig. 5B, magenta). The Na^+^ and Ca^2+^ cations in the major groove are negligible, whereas small fractions of SPD^3+^, PLL^6+^ and protamine clamp in the major groove (Fig. 5A, green), generating some disparity in major groove width (Fig. 5B, green). In addition, the minor-groove clamping cations make the minor groove of dsDNA more compact and less fluctuating (Table S1).

In summary, due to DNA condensation, commonly used HRM assays, whether dye-based or probe-based, are incapable of determining the effects of multivalent cations (*e.g.*, SPD^3+^, SM^4+^, PLL^6+^ and protamine) on DNA melting. We solved the problem by stretching DNA to induce melting at high force, at which DNA condensation is prevented (Fig. 2). By measuring the equilibrium unpeeling force and the corresponding changes in DNA extension, we determined the chemical free energy change during DNA unpeeling under each salt condition (Fig. 3A). We observed a clear non-monotonic dependence of DNA duplex stability on multivalent cation concentration, which may arise from charge inversion indicated by the concentration for each type of salt. At lower concentrations, cations stabilize dsDNA by neutralizing backbone charges and reducing electrostatic repulsion, whereas at higher concentrations they overcharge the duplex and increase electrostatic repulsion, leading to destabilization.^57,60–62^

As shown in Fig. 3C, another major finding is that the maximum DNA duplex stability increases monotonically with cation valency (Na^+^ < Ca^2+^ < SPD^3+^ < SM^4+^ < PLL^6+^ < protamine). Recent theoretical modeling and atomistic MD simulations further showed that cation valency and size affect DNA–cation interactions, while cell-like environments can alter the structure and dynamics of dsDNA.^63–66^ In the present simulations, we further resolved the distributions of cations within the grooves. Higher-valent cations more frequently formed minor-groove-associated interstrand clamping contacts, accompanied by minor-groove narrowing and reduced groove-width fluctuations, whereas major-groove clamping was less frequent. Previous atomistic simulations also revealed binding preferences of putrescine^2+^, SPD^3+^, SM^4+^, and protamine to DNA.^67,68^ These analyses support the same qualitative picture that the type, concentration, and valency of cations determine DNA duplex stability.

Higher-valent cations show stronger groove-associated localization (Fig. 5), consistent with the larger maximal duplex stabilization observed experimentally (Fig. 3C). Our all-atom MD simulations provide a structural mechanism for this trend: cations embed in DNA grooves and form “clamping” contacts that engage both strands (Fig. 4 and 5). The fraction of clamping cations increases strongly with valency and clamping cations occur mainly within the minor groove (Fig. 5A), in contrast to dsRNA, in which cation clamping preferentially occurs in the major groove.^60,61^ Minor-groove clamping represents a local electrostatic bridging mode that may stabilize the duplex beyond the nonspecific charge screening typically represented by monovalent salts. Correspondingly, increasing valency narrows the grooves, especially the minor groove, and yields a more compact, less fluctuating duplex (Fig. 5B and Table S1). These results suggest that multivalent cations stabilize dsDNA beyond the traditional diffuse electrostatic screening model:^59^ while Na^+^ and Ca^2+^ mainly screen phosphate charges, multivalent cations embed in grooves and clamp the duplex, strengthening DNA duplex stability more than screening alone. This finding is complementary to previous studies showing that SPD^3+^ and SM^4+^ attract different DNA duplexes beyond electrostatic screening.^11,14^

Beyond establishing a quantitative framework for DNA duplex stability across cation valencies, this work highlights the biological relevance of multivalent ionic environments in regulating nucleic acid structure and function. *In vivo*, DNA resides in crowded and heterogeneous milieus enriched with polyamines and arginine-rich proteins rather than simple monovalent salts. Our results show that DNA duplex stability is not set solely by bulk electrostatic screening, but can be tuned by ion valency and local binding modes. This provides a physical basis for how cells may modulate DNA accessibility, compaction, and mechanical robustness during transcription, replication, chromatin remodeling, and stress responses. More broadly, multivalent ions push nucleic acids into regimes in which ion correlations and local binding geometry become important, and condensation can readily mask intrinsic duplex thermodynamics (Fig. 1 and S5–S8). By using high tension to suppress condensation while keeping duplex melting accessible at equilibrium, our approach separates these coupled processes and provides an intrinsic thermodynamic benchmark under complex ionic conditions. These insights provide actionable constraints for designing ion-tunable DNA nanostructures and delivery systems and motivate future maps of stability in mixed-ion, crowded, and mechanically stressed environments.

## Conclusions

Multivalent cations play important roles in modulating the DNA structure, stability, and function. They can induce DNA condensation, compacting it from an extended coil into a dense structure. High-resolution melting is a commonly used tool for analyzing DNA duplex stability. However, high-resolution melting analysis is not well suited for characterizing multivalent cation systems because DNA condensation obscures the intrinsic duplex stability (Fig. 1 and S5–S8).

Together with previous studies of polyamine–DNA and salt–DNA interactions,^10,13–16,26^ we developed a high-tension single-molecule force-unpeeling approach that prevents DNA condensation and determines the effect of multivalent cations on DNA duplex stability. Our experimental data demonstrate the valency-dependent changes in DNA duplex stability, while MD simulations suggest a possible structural basis beyond the diffuse charge screening represented by monovalent salts.

Our main contributions are as follows: (i) we demonstrate a biphasic dependence of DNA duplex stability on cation concentration, which is consistent with DNA charge inversion. This biphasic dependence is observed for monovalent (Na^+^), divalent (Ca^2+^), and multivalent cations including SPD^3+^, SM^4+^, PLL^6+^, and protamine (∼21+). (ii) We show that the maximum DNA duplex stability increases with cation valence (from 1+ to 21+), suggesting a potential strategy for further stabilizing DNA in applications. (iii) All-atom molecular dynamics simulations uncover the mechanism underlying these observations: higher-valent cations more frequently form minor-groove-associated inter-strand clamping contacts, effectively attracting both strands and increasing duplex stability. These findings are broadly relevant to DNA–protein interactions, nucleic acid nanotechnology, and biomolecular electrostatics.

## Author contributions

Conceptualization: Y.-L. C. and X.-H. Z.; methodology: Y.-L. C. and X.-H. Z.; investigation: Y.-L. C. and C.-C. Z.; visualization: Z.-J. T. and X.-H. Z.; supervision: Y. Z., Z.-J. T. and X.-H. Z.; writing – original draft: Y.-L. C. and C.-C. Z.; writing – review and editing: Y.-L. C., Z.-J. T. and X.-H. Z.

## Conflicts of interest

There are no conflicts to declare.

## Supplementary Material

SC-OLF-D6SC03808E-s001

## Data Availability

The data supporting this article have been included as part of the supplementary information (SI). Supplementary information is available. See DOI: https://doi.org/10.1039/d6sc03808e.
