# Supplementary material for: Multivalent cations stabilize DNA duplexes beyond charge neutralization
Source: Chem Sci. 2026 Sep 1;17(41):19978–86. doi: 10.1039/d6sc03808e (PMC13552422; doi:10.1039/d6sc03808e)
Supplement: Supplementary file 1 [file SC-017-D6SC03808E-s001.pdf]

Supplementary Information (SI) for Chemical Science.
This journal is © The Royal Society of Chemistry 2026

Supplementary Materials for

Multivalent cations stabilize DNA duplexes beyond charge neutralization

Yun-Long Chen et al.

Corresponding authors: peneyyan@whu.edu.cn (Y. Zhang); zjtan@whu.edu.cn (Z.J. Tan); zhxh@whu.edu.cn (X.H. Zhang).

This PDF file includes:

Figures S1 to S9
Table S1
Methods S1 to S2

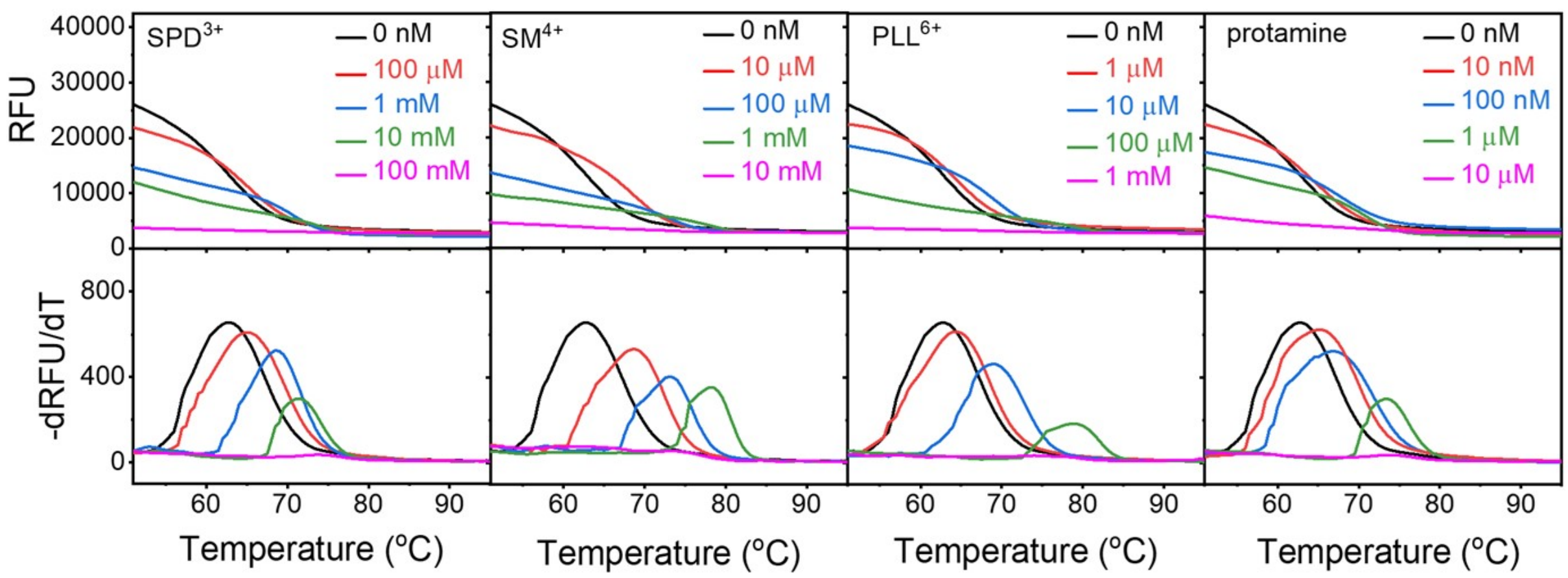

Figure S1. Representative melting curves and the negative first derivatives (-dRFU/dT) for the DNA at different concentrations of high-valent cations using dye assays. (A) SPD^{3+}. (B) SM^{4+}. (C) PLL^{6+}. (D) protamine.

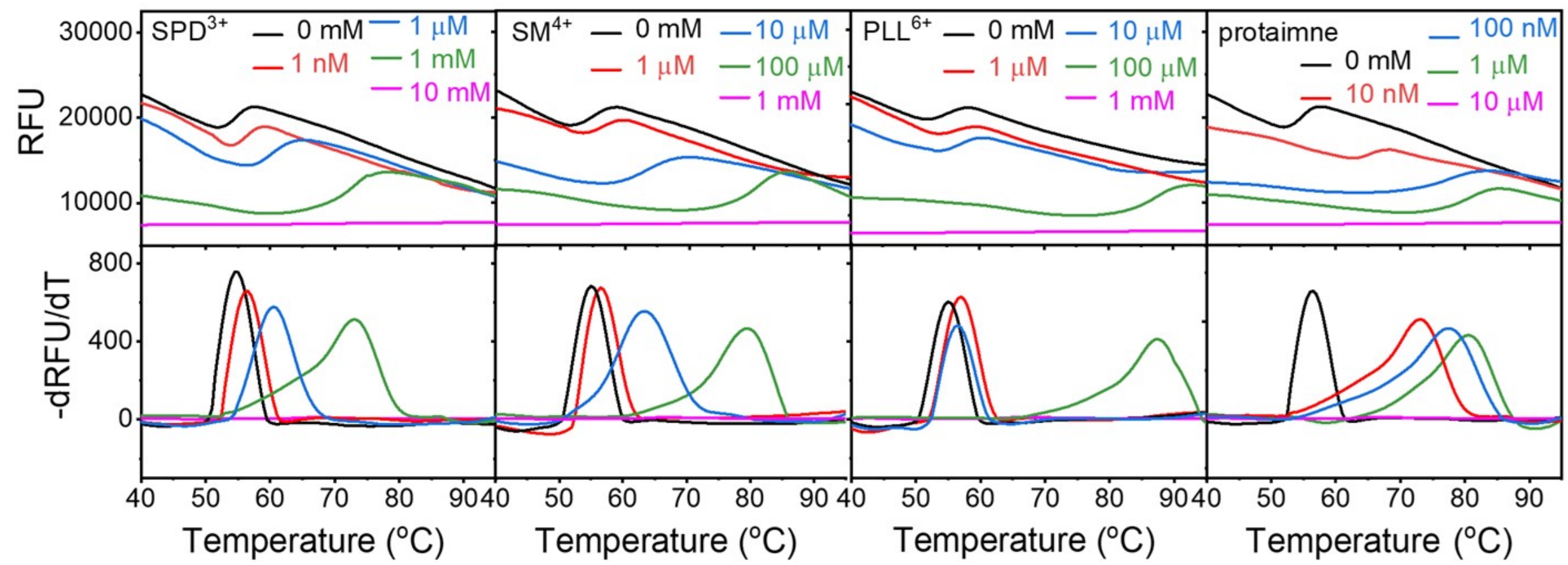

Figure S2. Representative melting curves and the negative first derivatives (-dRFU/dT) for the DNA at different concentrations of high-valent cations using probe assays. (A) SPD^{3+}. (B) SM^{4+}. (C) PLL^{6+}. (D) protamine.

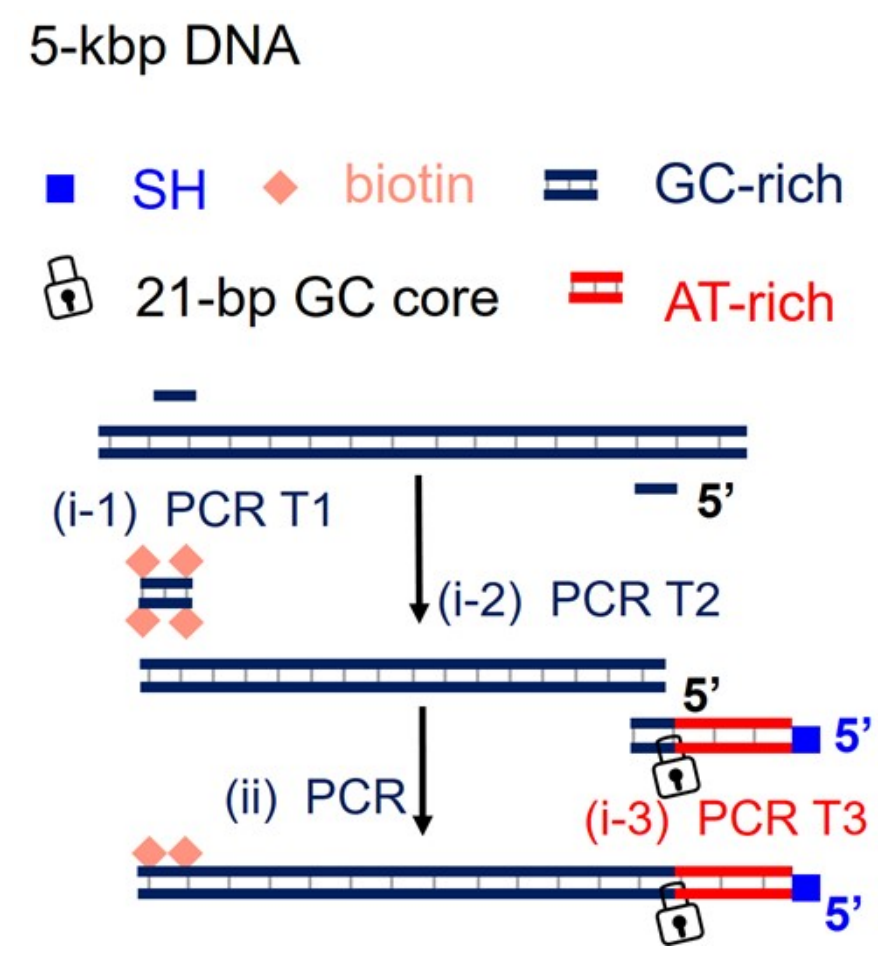

Figure S3. Preparation of DNA construct.

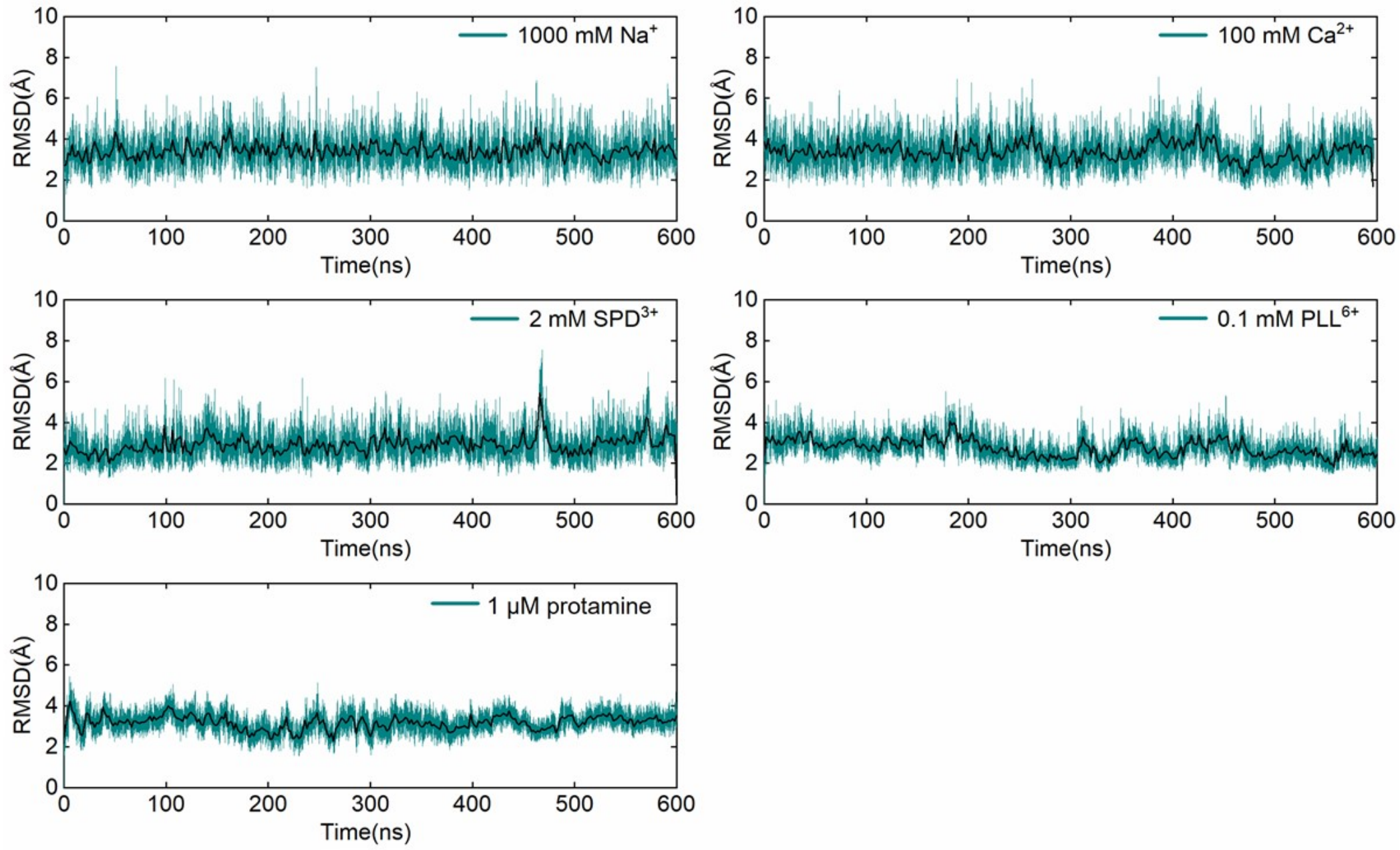

Figure S4. The root-mean-square deviation (RMSD) versus MD running time (600 ns) for dsDNA from a series of MD simulations under different ion conditions. The central black lines represent the RMSD values averaged over 2-ns intervals.

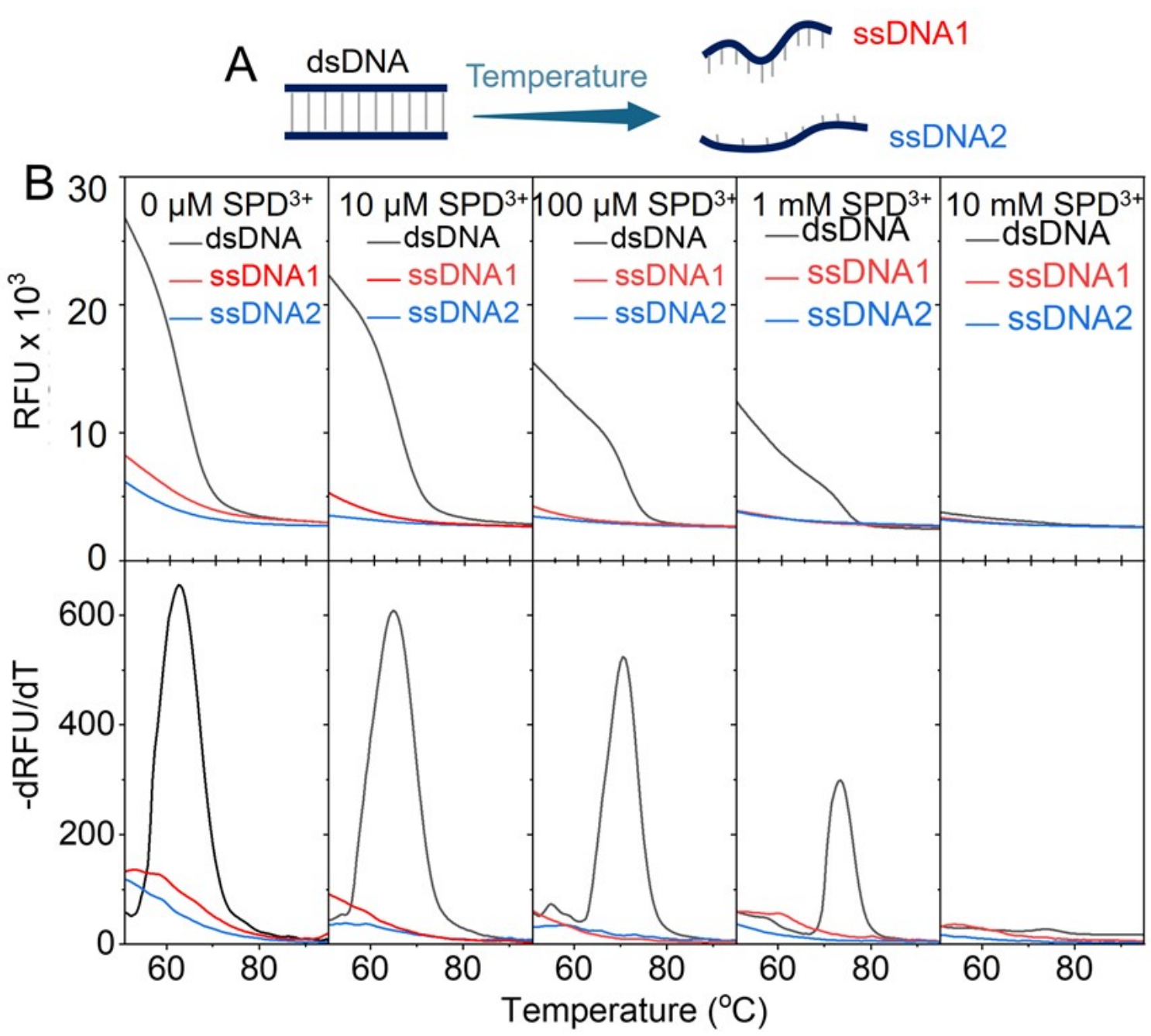

Figure S5. Representative fluorescence response curves and the negative first derivatives (-dRFU/dT) for the dsDNA and ssDNA at different SPD^{3+} concentrations using HRM experiments. (A) Schematic diagram showing dsDNA melting into two separate ssDNA strands. (B) Representative melting curves and negative first derivatives (-dRFU/dT) for dsDNA and ssDNA at different SPD^{3+} concentrations.

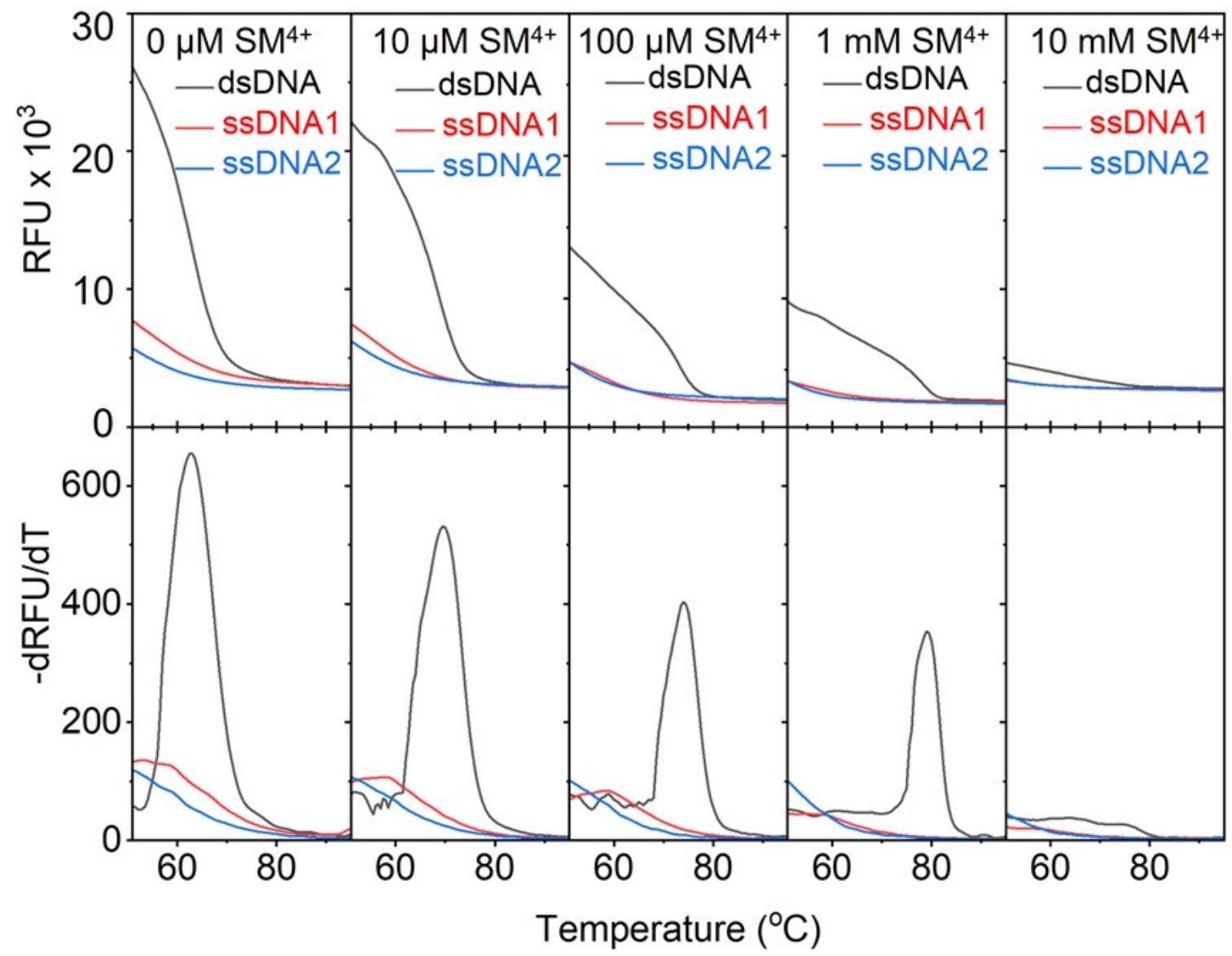

Figure S6. Representative fluorescence response curves and the negative first derivatives (-dRFU/dT) for the dsDNA and ssDNA at different SM^{4+} concentrations using HRM experiments.

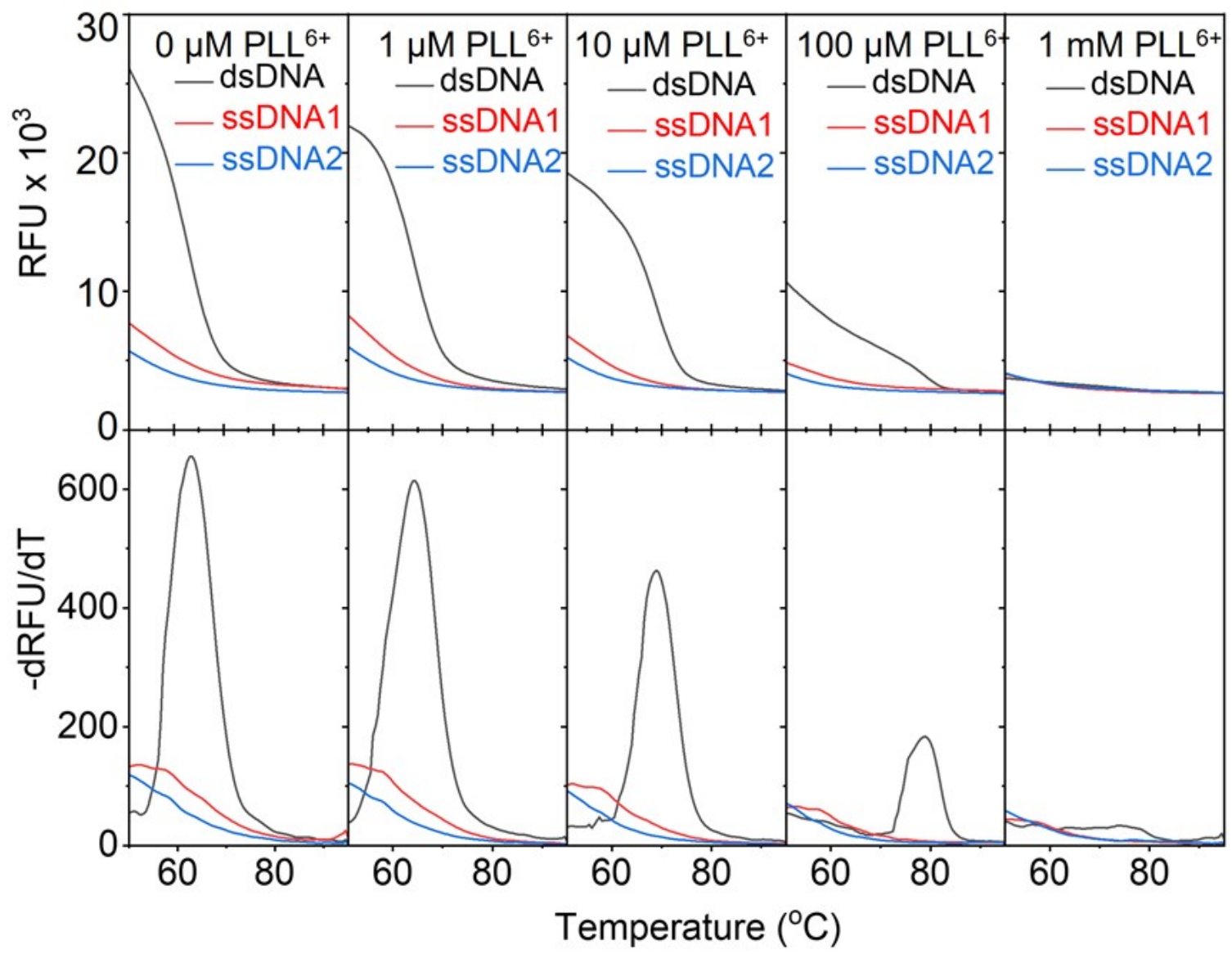

Figure S7. Representative fluorescence response curves and the negative first derivatives (-dRFU/dT) for the dsDNA and ssDNA at different PLL^{6+} concentrations using HRM experiments.

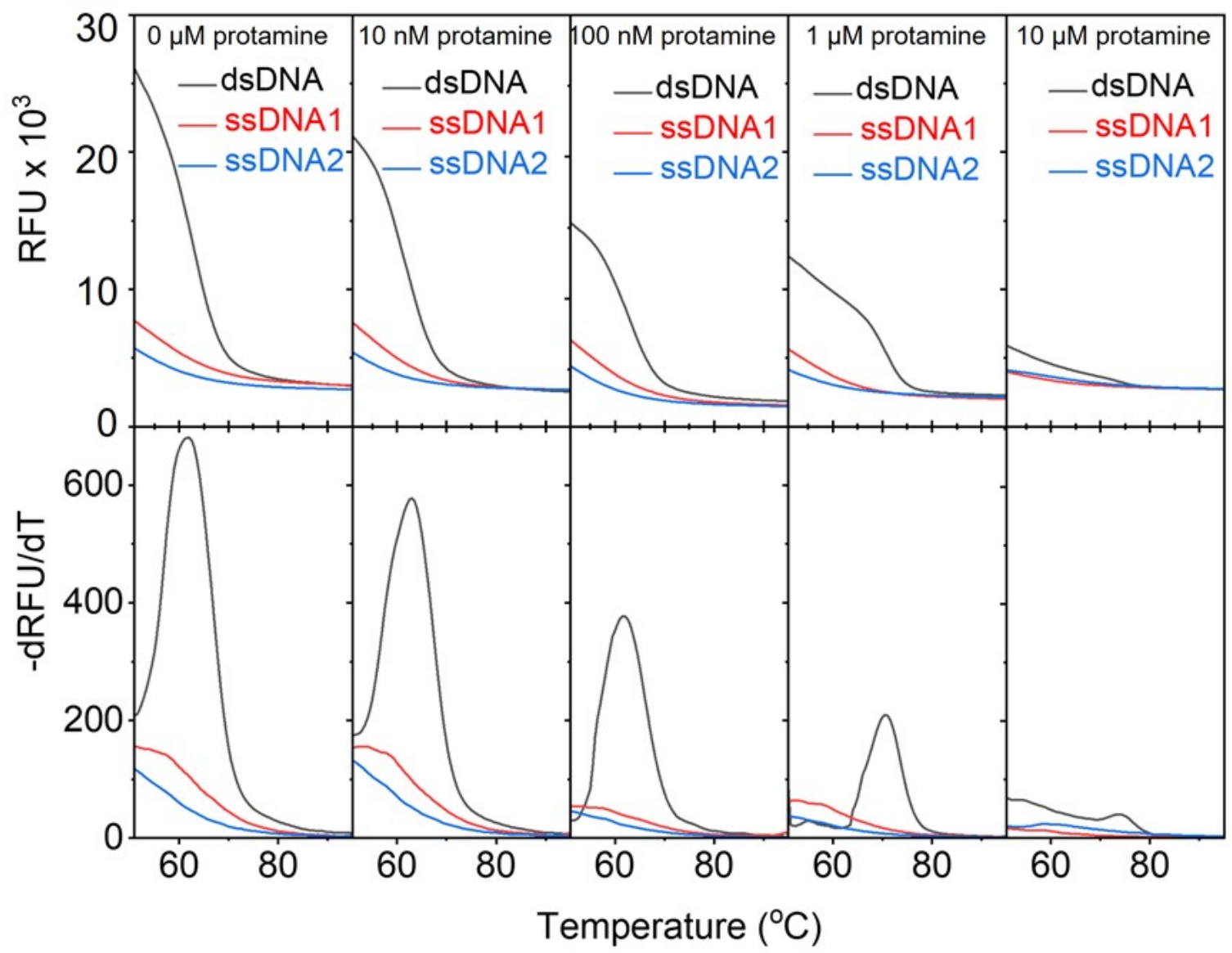

Figure S8. Representative fluorescence response curves and the negative first derivatives (-dRFU/dT) for the dsDNA and ssDNA at different protamine concentrations using HRM experiments.

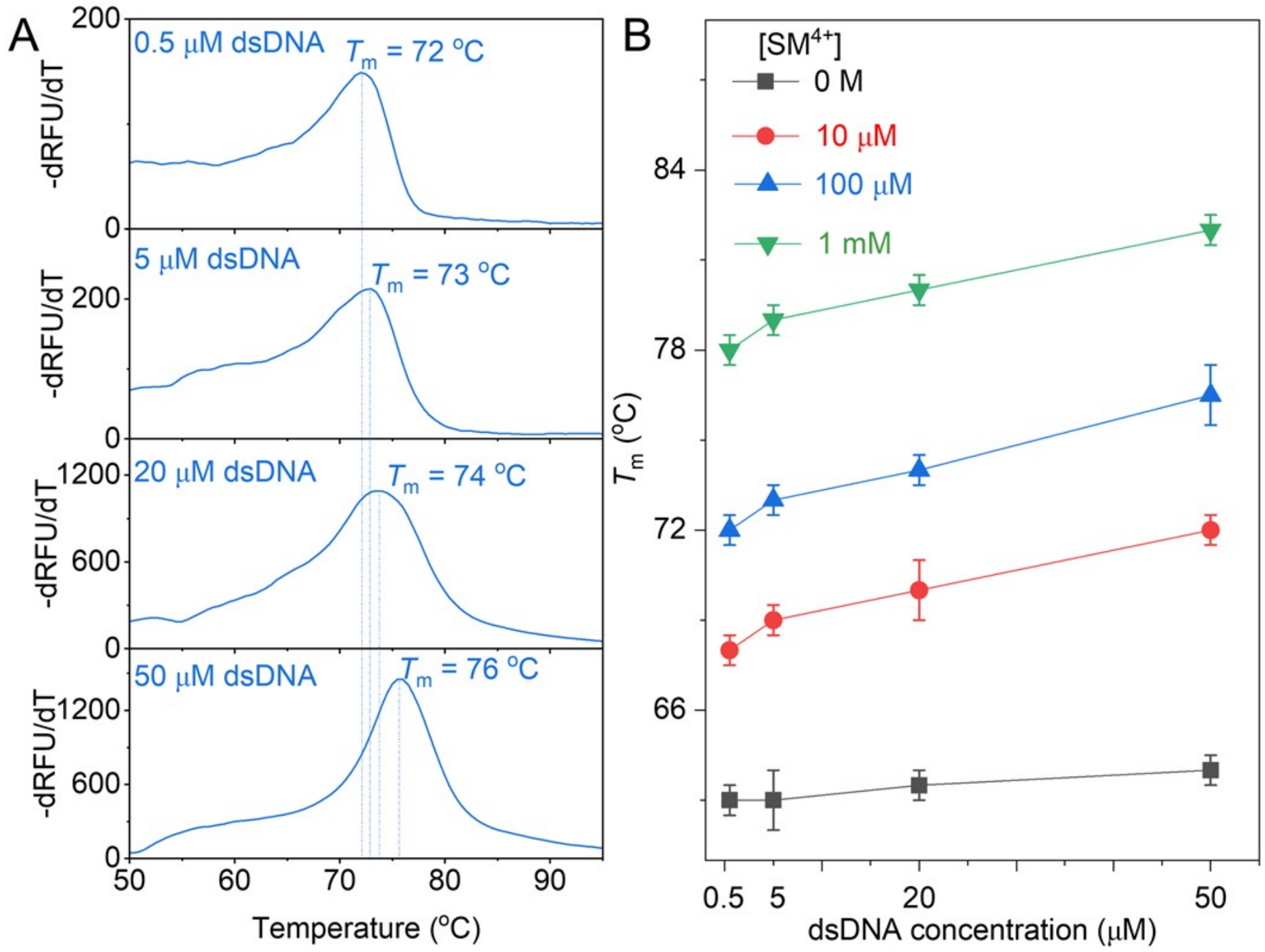

Figure S9. T_m versus dsDNA concentration at different SM^{4+} concentrations. (A) The negative first derivatives (-dRFU/dT) at different dsDNA concentrations in 100 μM SM^{4+}. (B) T_m as a function of dsDNA concentration at different SM^{4+} concentrations.

Table S1. Fluctuations in the minor- and major-groove widths. Δw^{minor} and Δw^{major} denote the standard deviations of the minor- and major-groove widths, respectively.

Parameters	Na^{+}	Ca^{2+}	SPD^{3+}	PLL^{6+}	protamine
Δw^{minor}(Å)	0.59	0.55	0.54	0.49	0.30
Δw^{major}(Å)	0.81	0.79	0.67	0.51	0.44

Method S1: Preparation of DNA for MT experiments

The detailed procedure for preparing DNA construct (Figure S3) is described below.

(i-1) Make a multiple-biotin-labeled short DNA fragment T1 (19000-19500 bp of lambda DNA) by PCR. In the PCR, F and Rs were primers and lambda DNA was the template. 30% biotin-11-dUTP was used to incorporate biotin groups into the PCR product (33).

(i-2) Make the 3.8-kbp DNA T2 (19000-22793 bp of lambda DNA) by PCR using F and R1 as primers and lambda DNA as template.

(i-3) Make the 700-bp short DNA fragment T3 (22800-23500 bp of lambda DNA) by PCR using F1 and R_SH as primers and lambda DNA as template.

(ii) Make the 5-kbp DNA by PCR using T1 and T3 as megaprimers and T2 as template.

All the oligos were synthesized in Sangon Biotech with the following sequences.

F: CGCACTTGCGGTGACAGTCACTCC

Rs: ACGGACGCTGCCGGATTAATCTGT

R_SH: SH-AACTGAATCATCTATTATTAAATCAGAAAGACG

F1: GTTGATATTTCTAAAGTCGGCCCGGGGGCCCCCGGCGGTTTTTTTCTTCGTTTTCTCT

R1: CCGACTTTAGAAATATCAACAGCAGACTCAAAACATGACGA

Method S2: Details of MD simulations

Force fields, ions, and run details

We performed a series of all-atom MD simulations at salt conditions including 1000 mM Na^+, 100 mM Ca^{2+}, 2 mM SPD^{3+}, 0.1 mM PLL^{6+} and 1 μM salmon protamine (UNIPROT P69014) (40). The initial structure of protamine was generated using the sequence command in the tleap module of AMBER (41-42). The structure was equilibrated in explicit solvent with 150 mM NaCl for 100 ns, and the equilibrated conformation was selected as the initial structure for subsequent simulations. Firstly, we constructed a 20-bp dsDNA with the sequence of 5'-CGACTCTACGGCATCTGCGC-3' using the Nucleic Acid Builder package (34). The dsDNA was solvated in a rectangular box containing explicit water and ions, which was large enough to guarantee a minimum distance of at least 12 Å from the dsDNA to the periodic box boundary. Subsequently, we added the ions of Na^+ and Cl^- with the model from Joung and Cheatham (35-36), Ca^{2+} with the Åquist model (37-38), SPD^{3+} and PLL^{6+} with the general AMBER force field (41), and protamine with the ff14SB force field (42), respectively, to neutralize the negatively charged dsDNA. Afterwards, we performed MD simulations using the Gromacs 2021.5 software package (43) with AMBER ff99bsc1 force fields for dsDNA (44) and a TIP3P water model (45-46) for molecular interactions. A cutoff of 10 Å was used for short-range electrostatic interactions and the particle mesh Ewald method for the long-range Coulombic interactions (47).

In each simulation, the systems were energy-minimized to eliminate any possible clashes and bad contacts. Then, an NVT ensemble was carried out for 2 ns with the V-rescale temperature coupling, and the systems were heated to 298 K. Subsequently, an NPT ensemble was conducted for 2 ns with Parrinello-Rahman pressure coupling to enable the pressure at 1 atm. During the pre-equilibration NVT and NPT periods, the dsDNA was restrained by a harmonic potential with a

force constant of 1000 kJ/(mol·nm^2). Finally, we performed 600-ns simulations for each system at different cation conditions with no restraints for the dsDNA. An integration step of 2 fs was applied with leap-frog, and the LINCS algorithm was used to constrain bonds between heavy atoms and hydrogens. According to Figure S4, the equilibriums were reached after ~200 ns, and the last 400-ns MD trajectories were used for the analyses. As for protamine, the chain was divided into three segments, each containing approximately 11 residues, and the interactions of each segment with DNA were analyzed separately using the same distance criterion.

Calculations of cation binding distributions around dsDNA

Based on the MD trajectories, we analyzed the major helical parameters and central helical axis using Curves+, including widths of major groove and minor groovea (52). In our analyses, we removed three base pairs at each end of the dsDNA to avoid the end effect (48-51). Furthermore, to elucidate the stabilization of high-valent cations on dsDNA, we calculated the cation binding distributions around dsDNA at different cation conditions. Specifically, we calculated the binding cations and clamping cations for dsDNA: (i) the binding cations were calculated as those cations around DNA within a radial distance of 6 Å (49-50); (ii) the clamping cations were calculated as those cations inside grooves and simultaneously interacting with two strands within a distance of 6 Å (53-56). These clamping cations can be further classified as major- and minor-groove clamping cations based on their locations in the major and minor grooves.
